# Prediction of metabolic and pre-metabolic syndromes using machine learning models with anthropometric, lifestyle, and biochemical factors from a middle-aged population in Korea

**DOI:** 10.1186/s12889-022-13131-x

**Published:** 2022-04-06

**Authors:** Junho Kim, Sujeong Mun, Siwoo Lee, Kyoungsik Jeong, Younghwa Baek

**Affiliations:** KM Data Division, Korea Institute of Oriental Medicine, 1672 Yuseongdae-ro, Yuseong-gu, Daejeon, Republic of Korea

**Keywords:** Metabolic syndrome, Machine learning, Feature importance, Data sampling method, SMOTE

## Abstract

**Background:**

Metabolic syndrome (MetS) is a complex condition that appears as a cluster of metabolic abnormalities, and is closely associated with the prevalence of various diseases. Early prediction of the risk of MetS in the middle-aged population provides greater benefits for cardiovascular disease-related health outcomes. This study aimed to apply the latest machine learning techniques to find the optimal MetS prediction model for the middle-aged Korean population.

**Methods:**

We retrieved 20 data types from the Korean Medicine Daejeon Citizen Cohort, a cohort study on a community-based population of adults aged 30–55 years. The data included sex, age, anthropometric data, lifestyle-related data, and blood indicators of 1991 individuals. Participants satisfying two (pre-MetS) or ≥ 3 (MetS) of the five NECP-ATP III criteria were included in the MetS group. MetS prediction used nine machine learning models based on the following algorithms: Decision tree, Gaussian Naïve Bayes, K-nearest neighbor, eXtreme gradient boosting (XGBoost), random forest, logistic regression, support vector machine, multi-layer perceptron, and 1D convolutional neural network. All analyses were performed by sequentially inputting the features in three steps according to their characteristics. The models’ performances were compared after applying the synthetic minority oversampling technique (SMOTE) to resolve data imbalance.

**Results:**

MetS was detected in 33.85% of the subjects. Among the MetS prediction models, the tree-based random forest and XGBoost models showed the best performance, which improved with the number of features used. As a measure of the models’ performance, the area under the receiver operating characteristic curve (AUC) increased by up to 0.091 when the SMOTE was applied, with XGBoost showing the highest AUC of 0.851. Body mass index and waist-to-hip ratio were identified as the most important features in the MetS prediction models for this population.

**Conclusions:**

Tree-based machine learning models were useful in identifying MetS with high accuracy in middle-aged Koreans. Early diagnosis of MetS is important and requires a multidimensional approach that includes self-administered questionnaire, anthropometric, and biochemical measurements.

## Background

Metabolic syndrome (MetS) is a complex condition that appears as a cluster of metabolic abnormalities, including obesity, hyperglycemia, hypertension, and dyslipidemia [1]. The global prevalence of MetS is 25–35% and increasing steadily [2–4]. The prevalence of MetS in the US increased from 25.3% in 1994–1998 to 34.2% in 2007–2012 (over 35% increase) [5]. The prevalence among the elderly remains high, while it is rapidly increasing among young adults [4]. During a similar period, the prevalence of MetS in Korea increased from 24.9% in 1998 to 31.3% in 2007 (25% increase) [6]. Furthermore, MetS is known to be a risk factor for increased morbidity and mortality associated with cardiovascular disease (CVD) or cancer [7, 8]. Detection before the MetS onset, treatment, and prevention are essential. For middle-aged individuals with no particular health issues, detecting the risk of MetS and active and healthy lifestyle intervention offer greater benefits for CVD-related health outcomes [9]. Change in systolic blood pressure, a major indicator of MetS, showed a strong association with CVD and all-cause deaths within the first 10 years of follow-up in males aged 40–59 years, weakening thereafter [10]. This highlights the importance of early detection of changes in the MetS components to facilitate early prediction of the disease in the middle-aged population.

Data mining, such as machine learning techniques, plays an important role in understanding the non-linear and complex relationships between various factors by extracting useful information that could help decision-making based on big data [11, 12]. In the medical field, machine learning techniques could analyze expansive clinical, imaging, and genomic data to improve the diagnostic and classification accuracy of diseases while presenting a new paradigm in treatment [13]. A previous study attempted to predict the onset of diabetes using data on risk factors of MetS collected over 10 years from 660,000 subjects [14]. In that study, Naïve Bayes and J48 decision tree decision-making models and various machine learning techniques were shown to be optimal for predicting diabetes. The effect of various sampling techniques was also verified. Moreover, a recent study attempted to use machine learning based on various clinical features to predict MetS [15, 16]. A MetS prediction model for a working population using an artificial neural network was highly efficient, with an accuracy of 89%, higher than logistic regression analysis, the traditional prediction modeling technique [16]. Moreover, in a study that used the Naïve Bayes model to predict MetS based on clinical and genetic data of a normal-weight population, the area under the receiver operating characteristic curve (AUC) increased by 4% when genetic feature composed of single nucleotide polymorphism was added to the baseline clinical feature such as gender and age, indicating the importance of feature selection [15].

In a systematic review that analyzed 22 reports on MetS prediction in the Korean population during the past 10 years [17], the study population in most consisted of all age groups, including the elderly, rather than any specific age group population. The most used MetS diagnostic criteria were those in the National Cholesterol Education Program-Adult Treatment Panel III (NCEP-ATP III) guidelines. Moreover, 64% of the studies used logistic regression analysis to predict MetS. One study used five machine learning MetS prediction models, with eXtreme gradient boosting (XGBoost) (AUC = 0.879) showing the best performance [18]. There are almost no studies on MetS machine learning prediction based on various clinical features in middle-aged Koreans.

The objective of the present study was to construct an optimal MetS prediction model by applying machine learning techniques to data pertaining to middle-aged Koreans. This study also performed MetS prediction modeling by including pre-MetS (at least two components of the MetS diagnostic criteria) for preventive healthcare of the study population. We assessed the contribution degree of the various clinical feature types and examined the model performance changes after applying data sampling to minimize data imbalance.

## Methods

### Study design and participants

This study used data from the community-based Korean Medicine Daejeon Citizen Cohort (KDCC) study currently undergoing in Korea [19]. The KDCC study includes 30–55 years old residents of Daejeon, excluding individuals diagnosed with cancer or CVD (myocardial infarction, angina, stroke/apoplexy). The study completed a population-based survey of 2000 participants between 2017 and 2019 to collect demographic, lifestyle-related, individual characteristics of Korean medicine (KM), clinical, and biochemical measurements data. The questionnaire survey was conducted as a face-to-face interview by well-trained interviewers. The participants height, weight, waist circumference, and hip circumference were measured. Samples for blood tests, collected after 12 h of fasting, were sent for testing to an authorized diagnostic laboratory (Seoul Clinical Laboratories, Seoul, Korea). This study analyzed the KDCC data of 1991 individuals after excluding nine with missing values.

The KDCC study was approved by the Institutional Review Board, and informed consent forms were obtained from the participants after providing an explanation about their participation in the study.

### Measures

With reference to previous studies, the 20 features used in the MetS prediction models were examined [17] and added sequentially in three steps [15, 16] taking into account their characteristics and methods of collection: demographic and anthropometric data that could be self-reported or were already known were added in step 1; lifestyle-related factors that could be measured using questionnaires were added in step 2; and blood indicators were added in step 3. The variables used in this study are well known risk factors for metabolic syndrome in the clinical setting. In addition, these variables are important modifiable factors through clinical attention and individual intervention and awareness for the prediction and management of metabolic syndrome [1].

#### Demographic and body measurements (Step 1)

The first group of features consisted of sex, age, body mass index (BMI), and waist-to-hip ratio (WHR). BMI was calculated by dividing the measured weight (kg) by the squared height (m^2^), while WHR was calculated by dividing the average waist circumference by the average hip circumference after performing two measurements for each with a measuring tape (Rollfix, Hoechstmass, Germany).

#### Lifestyle-related factors (Step 2)

The second group of features consisted of lifestyle-related factors, including drinking status, smoking status, physical activity [20], sleep time and quality [21], eating index [22], stress [23], and symptom-based KM types used [24]. All eight features were investigated using a structured questionnaire. The following questions were asked for smoking status: “Have you smoked more than 100 cigarettes in your lifetime?” and “Do you currently smoke?” Based on the responses, the smoking status of the participants was classified as “current smoker,” “former smoker,” and “non-smoker.” Drinking status was classified as “current drinker,” “former drinker,” and “non-drinker” based on similar questions about drinking. Physical activity (PA) was assessed using the Korean Global Physical Activity Questionnaire developed by the World Health Organization [20]. PA was calculated and later converted to Metabolic equivalent of task (METs). The sleeping time and quality over the past month were assessed using the Korean version of the Pittsburgh Sleep Quality Index [25]. Eating index was measured using a semi-quantitative food frequency questionnaire consisting of 34 food groups, which collects data on the frequency (nine option ranging from rarely eaten to three times a day) and average intake (three or four specified portion sizes) of each food item over the past year [19]. Eating index was composed of 9 adequacy components and 5 moderate components, and the total score ranged from 0 to 100 following the previously-reported calculation method of the Korean Healthy Eating Index [26]. The stress index was calculated using the 18-item Psychosocial Well-being Index-Short Form [27]. The KM types were defined as Sasang constitution and were determined by the simplified and structured questionnaire comprised one physical characteristic, six personality traits, and eight physiological symptoms [24]. The KM types were classified into Taeumin, Soeumin, or Soyangin because users of these types vary in their physiological and psychological states, disease susceptibility, and lifestyle healthcare approach [28].

#### Biochemical measurements (Step 3)

The third group of features consisted of eight blood test features, including aspartate transaminase (AST), alanine transaminase (ALT), and alkaline phosphatase (ALP) for liver function [29]; high-sensitivity C-reactive protein (hsCRP) [30]; hemoglobin A1c (HbA1c) [31]; insulin; gamma-glutamyl transferase (GGT); and homeostatic model assessment for insulin resistance (HOMA-IR) [32]. Blood samples were collected from a peripheral vein in the morning, following overnight fasting, and then centrifuged at 3450 rpm for 10 min. Blood samples were examined using automatic clinical chemistry analyzers (ADVIA1800, Siemens, USA) for AST, ALT, ALP, hsCRP, and GGT, also including glucose, triglyceride, and high-density lipoprotein-cholesterol as diagnostic indicators of Mets. HbA1c and insulin levels were determined using an automated analyzer (Variant II trubo, BIORAD, USA and ADIVA Centaur, Siemens, USA, respectively). HOMA-IR was calculated as glucose (mg/dL) × insulin level (mIU/L)/405.

### Definition of the metabolic syndrome

The Mets group in present study was defined as meeting at least two criteria including both pre-MetS and Mets status, because of the importance of preventive healthcare by early detection of MetS in the middle-aged population [33]. MetS group was diagnosed by the following five criteria given in the NCEP-ATP III guidelines [1]: 1) a waist circumference above the cut-off point for Koreans (≥90 cm for males and ≥ 85 cm for females); 2) systolic blood pressure ≥ 130 mmHg, diastolic blood pressure ≥ 85 mmHg, or taking medication for hypertension; 3) a triglyceride level of ≥150 mg/dL or taking medication for such lipid abnormalities; 4) low high-density lipoprotein-cholesterol level (< 40 mg/dL for males and < 50 mg/dL for females) or taking medication for such lipid abnormalities; 5) a fasting plasma glucose level of ≥100 mg/dL or taking medication for type 2 diabetes.

### Analysis

Data are expressed as mean and standard deviation, and frequency and percentage. General characteristics of the participants between the normal and the Mets groups were compared by the Fisher’s exact test or chi-square test for categorical variables and by independent t-tests for continuous variables. The performance of the MetS prediction models was compared by sequentially inputting the 20 features identified as key indicators on MetS in three steps and examining their influence. A list and scale of features by stage are as follows. In step 1, sex as categorical variable, and age, BMI, and WHR as continuous variables were inputted. In step 2, drinking, smoking, KM types as categorical variables, and physical activity, sleep time, sleep quality, eating index, and stress as continuous variables were additionally inputted. In step 3, AST, ALT, ALP, hsCRP, HbA1c, insulin, GGT, and HOMA-IR as continuous variables were additionally inputted.

A supervised machine learning model was used for MetS prediction. The algorithms used to develop the model were decision tree, Gaussian Naïve Bayes (NB), K-nearest neighbor (KNN) [34], XGBoost, random forest (RF), logistic regression [15, 18, 35], support vector machine (SVM), multi-layer perceptron (MLP) [16], and 1-dimensional convolutional neural network (1D-CNN) [36]. Min-max normalization was applied to the data used in the analysis [37]. The model was built using 6-fold classified training data and test data. The ratio of the number of training and test datasets was 5:1. Of the 1991 datasets, 1659 and 332 datasets were used for training dataset, and test dataset, respectively. In addition, the 2:1 ratio of the normal group and the Mets group was configured to remain the same for the training and the test datasets. Moreover, we performed oversampling using the synthetic minority oversampling technique (SMOTE) to deal with data imbalance [13, 38, 39]. The SMOTE generates randomly synthesized data for the minority class using the Euclidean distance-based nearest neighbor approach. The synthesized and existing data had similar characteristics as the generation of the synthesized data was based on existing data. We compared the performances before and after the SMOTE application. Lastly, RF [18, 40] investigated the importance of features influencing the MetS. This is because the performance of the RF model consistently showed the best overall performance in all three stages.

The performance of the MetS prediction models was measured using F1-score, accuracy, sensitivity, specificity, and the AUC, along with 95% confidence interval. F1-score is the harmonic mean of precision and recall, and the calculation formula is as follows: F1-score = 2 / {(1/Precision) + (1/Recall)}, Precision = True Positive / (True Positive + False Positive), and Recall = True Positive / (True Positive + False Negative). Scikit-learn library in Python ver. 3.8.5 (Python Software Foundation, https://www.python.org/psf/) was used. For analysis and comparison, a model was built using default parameters.

## Results

### General characteristics

The characteristics of the 1991 participants (30.5% males and 69.5% females) are detailed in Table 1. Of these, 1317 were considered normal group, and 674 were considered to have Mets group. The differences in the characteristics of the variables between the normal and the Mets groups are shown in Table 1. There were significant differences in all variables except for age of 45 years or younger, physical activity, sleep time, sleep quality, eating index, and stress (Table 1).

**Table 1:** General participant characteristics

	Total	Normal group	MetS group	*p*-value
Sex	1991 (100)	1317 (66.1)	674 (33.9)	
Male	608 (30.5)	297 (48.8)	311 (51.2)	< 0.001
Female	1383 (69.5)	1020 (73.8)	363 (26.2)	
Age (years)	43.81 ± 6.86	43.12 ± 6.83	45.17 ± 6.73	< 0.001
30–44	1006 (50.5)	728 (72.4)	278 (27.6)	0.373
45–55	985 (49.5)	589 (59.8)	396 (40.2)	0.003
BMI (kg/m^2^)	24.34 ± 3.62	22.96 ± 2.79	27.05 ± 3.53	< 0.001
WHR	0.86 ± 0.06	0.84 ± 0.05	0.90 ± 0.05	< 0.001
Alcohol status				
non-drinker	716 (100)	500 (69.8)	216 (30.2)	0.007
former drinker	76 (100)	52 (68.4)	24 (31.6)	
current drinker	1199 (100)	765 (63.8)	434 (36.2)	
Smoking status				
non-smoker	1585 (100)	1122 (70.8)	463 (29.2)	< 0.001
former smoker	162 (100)	75 (46.3)	87 (53.7)	
current smoker	244 (100)	120 (49.8)	124 (50.8)	
KM type				
Taeumin	1012 (100)	492 (48.6)	520 (51.4)	< 0.001
Soeumin	397 (100)	351 (88.4)	46 (11.6)	
Soyangin	582 (100)	474 (81.4)	108 (18.6)	
PA (METs)	2538 ± 3798.53	2606.85 ± 39,258.16	2405.60 ± 3527.73	0.264
Sleep time (h)	6.71 ± 1.06	6.74 ± 1.04	6.66 ± 1.09	0.139
Sleep quality	4.69 ± 2.86	4.69 ± 2.81	4.68 ± 2.97	0.959
Eating index	51.42 ± 10.50	51.72 ± 10.46	50.82 ± 10.53	0.069
Stress	17.65 ± 7.07	17.60 ± 7.26	17.75 ± 6.69	0.657
AST (U/L)	24.89 ± 12.14	23.53 ± 10.06	27.55 ± 15.07	< 0.001
ALT (U/L)	23.97 ± 19.81	20.19 ± 14.78	31.35 ± 25.48	< 0.001
ALP (U/L)	63.70 ± 18.68	60.66 ± 17.68	69.62 ± 19.15	< 0.001
hsCRP (mg/L)	1.25 ± 2.76	1.00 ± 2.69	1.73 ± 2.85	< 0.001
HbA1c (%)	5.48 ± 0.60	5.35 ± 0.31	5.75 ± 0.87	< 0.001
Insulin (mIU/L)	6.10 ± 4.34	4.90 ± 2.90	8.45 ± 5.56	< 0.001
GGT (U/L)	30.18 ± 38.20	22.64 ± 27.18	44.92 ± 50.40	< 0.001
HOMA-IR	1.30 ± 1.14	0.99 ± 0.64	1.94 ± 1.57	< 0.001
Mets components				
Waist circumference (cm)	82.72 ± 9.67	78.92 ± 7.71	90.13 ± 8.76	< 0.001
Triglyceride (mg/dL)	132.31 ± 124.14	95.05 ± 50.38	205.12 ± 180.42	< 0.001
HDL-C (mg/dL)	56.87 ± 13.89	61.54 ± 12.97	47.74 ± 10.72	< 0.001
Systolic BP (mmHg)	116.97 ± 15.34	112.10 ± 12.47	126.49 ± 15.95	< 0.001
Diastolic BP (mmHg)	73.52 ± 12.08	69.75 ± 9.95	80.88 ± 12.48	< 0.001
Glucose (mg/dL)	84.16 ± 16.20	80.59 ± 8.00	91.14 ± 24.01	< 0.001

*MetS* Metabolic syndrome, *BMI* Body mass index, *WHR* Waist-to-hip ratio, *KM type* Korean medicine type, *PA* Physical activity, *METs* Metabolic equivalent of task, *AST* Aspartate transaminase, *ALT* Alanine transaminase, *ALP* Alkaline phosphatase, *hsCRP* High sensitivity C-reactive protein, *HbAlc* Hemoglobin A1c, *GGT* Gamma-glutamyl transferase, *HOMA-IR* Homeostatic model assessment for insulin resistance, *HDL-C* High-density lipoprotein-cholesterol, *BP* Blood pressure

Values are presented as n (%) or mean ± standard deviation

^†^*P*-values for continuous are based on independent t-tests; all other *P*-values for categorical variables are based on Fisher’s exact test or chi-square test between the normal and Mets groups

### Comparison of the machine learning models without the synthetic minority oversampling technique

When sex, age, BMI, and WHR were used in the nine MetS prediction models before applying SMOTE, the Gaussian NB model showed the highest AUC (range for all models, 0.677–0.764), sensitivity (range for all models, 0.558–0.684), and F1-score (range for all models, 0.711–0.789). When MetS was predicted with the addition of the 8 lifestyle-related features to the 4 features, the models had an AUC range of 0.686–0.756, sensitivity range of 0.551–0.685, and F1-score range of 0.722–0.791. The RF model showed the best performance in the AUC and F1 score. When all 20 features were used to predict MetS, the models had an AUC range of 0.703–0.786 and an F1-score range of 0.743–0.815, with the RF model showing the best performance based on AUC (0.786) and sensitivity (0.690). Some models, particularly the tree-based models, such as XGBoost and RF, tended to show improved performance with the increase in the number of features (Table 2).

**Table 2:** The models’ performance with 95% confidence interval according to the number of features used

	F1-score		Accuracy		Sensitivity		Specificity		AUC	
	Original	SMOTE	Original	SMOTE	Original	SMOTE	Original	SMOTE	Original	SMOTE
***4 Features (Demographic and anthropometric Features)***										
Decision Tree	0.711 (0.66–0.76)	0.758 (0.71–0.80)	0.711 (0.66–0.76)	0.758 (0.71–0.80)	0.573 (0.52–0.63)	0.758 (0.71–0.80)	0.782 (0.74–0.83)	0.758 (0.71–0.80)	0.677 (0.63–0.73)	0.758 (0.71–0.80)
Gaussian NB	0.789 (0.75–0.83)	0.780 (0.74–0.82)	0.790 (0.75–0.83)	0.780 (0.74–0.82)	0.684 (0.63–0.73)	0.790 (0.75–0.83)	0.844 (0.80–0.88)	0.769 (0.72–0.81)	0.764 (0.72–0.81)	0.780 (0.74–0.82)
KNN	0.774 (0.73–0.82)	0.783 (0.74–0.83)	0.777 (0.73–0.82)	0.783 (0.74–0.83)	0.619 (0.57–0.67)	0.826 (0.79–0.87)	0.859 (0.82–0.90)	0.740 (0.69–0.79)	0.739 (0.69–0.79)	0.783 (0.74–0.83)
XGBoost	0.771 (0.73–0.82)	0.802 (0.76–0.84)	0.773 (0.73–0.82)	0.802 (0.76–0.85)	0.626 (0.57–0.68)	0.812 (0.77–0.85)	0.848 (0.81–0.89)	0.792 (0.75–0.84)	0.737 (0.69–0.78)	0.802 (0.76–0.85)
RF	0.772 (0.73–0.82)	0.813 (0.77–0.86)	0.774 (0.73–0.82)	0.814 (0.77–0.86)	0.628 (0.58–0.68)	0.832 (0.79–0.87)	0.850 (0.81–0.89)	0.795 (0.75–0.84)	0.739 (0.69–0.79)	0.814 (0.77–0.86)
Logistic R	0.777 (0.73–0.82)	0.783 (0.74–0.83)	0.787 (0.74–0.83)	0.784 (0.74–0.83)	0.558 (0.50–0.61)	0.799 (0.76–0.84)	0.904 (0.87–0.94)	0.768 (0.72–0.81)	0.731 (0.68–0.78)	0.784 (0.74–0.83)
SVM	0.787 (0.74–0.83)	0.785 (0.74–0.83)	0.795 (0.75–0.84)	0.785 (0.74–0.83)	0.585 (0.53–0.64)	0.809 (0.77–0.85)	0.903 (0.87–0.93)	0.762 (0.72–0.81)	0.744 (0.70–0.79)	0.786 (0.74–0.83)
MLP	0.785 (0.74–0.83)	0.770 (0.72–0.82)	0.792 (0.75–0.84)	0.772 (0.73–0.82)	0.607 (0.55–0.66)	0.735 (0.69–0.78)	0.887 (0.85–0.92)	0.809 (0.77–0.85)	0.747 (0.70–0.79)	0.772 (0.73–0.82)
1D-CNN	0.779 (0.73–0.82)	0.783 (0.74–0.83)	0.782 (0.74–0.83)	0.784 (0.74–0.83)	0.657 (0.61–0.71)	0.784 (0.74–0.83)	0.846 (0.81–0.88)	0.784 (0.74–0.83)	0.752 (0.71–0.80)	0.784 (0.74–0.83)
***12 Features (Lifestyle-related features added)***										
Decision Tree	0.722 (0.67–0.77)	0.765 (0.72–0.81)	0.724 (0.68–0.77)	0.765 (0.72–0.81)	0.570 (0.52–0.62)	0.776 (0.73–0.82)	0.803 (0.76–0.85)	0.755 (0.71–0.80)	0.686 (0.64–0.74)	0.765 (0.72–0.81)
Gaussian NB	0.775 (0.73–0.82)	0.766 (0.72–0.81)	0.774 (0.73–0.82)	0.766 (0.72–0.81)	0.685 (0.64–0.74)	0.773 (0.73–0.82)	0.820 (0.78–0.86)	0.759 (0.71–0.81)	0.753 (0.71–0.80)	0.766 (0.72–0.81)
KNN	0.738 (0.69–0.78)	0.780 (0.73–0.82)	0.743 (0.70–0.79)	0.782 (0.74–0.83)	0.551 (0.50–0.60)	0.879 (0.84–0.91)	0.842 (0.80–0.88)	0.685 (0.63–0.73)	0.696 (0.65–0.75)	0.782 (0.74–0.83)
XGBoost	0.778 (0.73–0.82)	0.834 (0.79–0.87)	0.782 (0.74–0.83)	0.834 (0.79–0.87)	0.622 (0.57–0.67)	0.837 (0.8–0.88)	0.863 (0.83–0.90)	0.832 (0.79–0.87)	0.743 (0.70–0.79)	0.834 (0.79–0.87)
RF	0.791 (0.75–0.83)	0.838 (0.80–0.88)	0.795 (0.75–0.84)	0.838 (0.80–0.88)	0.635 (0.58–0.69)	0.850 (0.81–0.89)	0.876 (0.84–0.91)	0.826 (0.79–0.87)	0.756 (0.71–0.80)	0.838 (0.80–0.88)
Logistic R	0.785 (0.74–0.83)	0.779 (0.73–0.82)	0.792 (0.75–0.84)	0.779 (0.73–0.82)	0.595 (0.54–0.65)	0.791 (0.75–0.83)	0.893 (0.86–0.93)	0.767 (0.72–0.81)	0.744 (0.70–0.79)	0.779 (0.73–0.82)
SVM	0.790 (0.75–0.83)	0.783 (0.74–0.83)	0.797 (0.75–0.84)	0.783 (0.74–0.83)	0.605 (0.55–0.66)	0.796 (0.75–0.84)	0.894 (0.86–0.93)	0.770 (0.72–0.82)	0.750 (0.70–0.80)	0.783 (0.74–0.83)
MLP	0.772 (0.73–0.82)	0.797 (0.75–0.84)	0.778 (0.73–0.82)	0.798 (0.75–0.84)	0.619 (0.57–0.67)	0.790 (0.75–0.83)	0.859 (0.82–0.90)	0.806 (0.76–0.85)	0.739 (0.69–0.79)	0.798 (0.75–0.84)
1D-CNN	0.771 (0.73–0.82)	0.770 (0.72–0.82)	0.776 (0.73–0.82)	0.774 (0.73–0.82)	0.635 (0.58–0.69)	0.861 (0.82–0.90)	0.848 (0.81–0.89)	0.688 (0.64–0.74)	0.742 (0.69–0.79)	0.775 (0.73–0.82)
***20 Features (Biochemical measurements added)***										
Decision Tree	0.743 (0.70–0.79)	0.777 (0.73–0.82)	0.743 (0.70–0.79)	0.778 (0.73–0.82)	0.631 (0.58–0.68)	0.797 (0.75–0.84)	0.801 (0.76–0.84)	0.758 (0.71–0.80)	0.716 (0.67–0.76)	0.778 (0.73–0.82)
Gaussian NB	0.786 (0.74–0.83)	0.759 (0.71–0.81)	0.795 (0.75–0.84)	0.762 (0.72–0.81)	0.577 (0.52–0.63)	0.646 (0.59–0.70)	0.906 (0.87–0.94)	0.878 (0.84–0.91)	0.741 (0.69–0.79)	0.762 (0.72–0.81)
KNN	0.748 (0.70–0.79)	0.787 (0.74–0.83)	0.756 (0.71–0.80)	0.788 (0.74–0.83)	0.540 (0.49–0.59)	0.871 (0.83–0.91)	0.866 (0.83–0.90)	0.705 (0.66–0.75)	0.703 (0.65–0.75)	0.788 (0.74–0.83)
XGBoost	0.801 (0.76–0.84)	0.851 (0.81–0.89)	0.804 (0.76–0.85)	0.851 (0.81–0.89)	0.662 (0.61–0.71)	0.859 (0.82–0.9)	0.877 (0.84–0.91)	0.843 (0.8–0.88)	0.769 (0.72–0.81)	0.851 (0.81–0.89)
RF	0.815 (0.77–0.86)	0.843 (0.80–0.88)	0.818 (0.78–0.86)	0.844 (0.80–0.88)	0.690 (0.64–0.74)	0.857 (0.82–0.89)	0.883 (0.85–0.92)	0.831 (0.79–0.87)	0.786 (0.74–0.83)	0.844 (0.80–0.88)
Logistic R	0.812 (0.77–0.85)	0.804 (0.76–0.85)	0.818 (0.78–0.86)	0.804 (0.76–0.85)	0.638 (0.59–0.69)	0.812 (0.77–0.85)	0.910 (0.88–0.94)	0.796 (0.75–0.84)	0.774 (0.73–0.82)	0.804 (0.76–0.85)
SVM	0.811 (0.77–0.85)	0.810 (0.77–0.85)	0.817 (0.78–0.86)	0.810 (0.77–0.85)	0.636 (0.58–0.69)	0.831 (0.79–0.87)	0.909 (0.88–0.94)	0.790 (0.75–0.83)	0.773 (0.73–0.82)	0.810 (0.77–0.85)
MLP	0.807 (0.76–0.85)	0.811 (0.77–0.85)	0.812 (0.77–0.85)	0.812 (0.77–0.85)	0.638 (0.59–0.69)	0.836 (0.80–0.88)	0.901 (0.87–0.93)	0.787 (0.74–0.83)	0.770 (0.72–0.81)	0.812 (0.77–0.85)
1D-CNN	0.799 (0.76–0.84)	0.814 (0.77–0.86)	0.803 (0.76–0.85)	0.815 (0.77–0.86)	0.662 (0.61–0.71)	0.807 (0.76–0.85)	0.875 (0.84–0.91)	0.822 (0.78–0.86)	0.768 (0.72–0.81)	0.815 (0.77–0.86)

Presented are the results before (Original) and after (SMOTE) applying the synthetic minority oversampling technique

*AUC* Area under the receiver operating characteristic curve, *Gaussian NB* Gaussian naïve bayes classifier, *KNN* K-nearest neighbor, *XGBoost* Extreme gradient boosting, *Logistic R* Logistic regression, *RF* Random forest, *SVM* Support vector machine, *MLP* Multilayer perceptron, *1D-CNN* 1-dimensional convolutional neural network

### Performances with the synthetic minority oversampling technique

Due to imbalance between the MetS group and normal group, the performance of the models was assessed before and after applying the SMOTE. When the MetS models were constructed with four features and the SMOTE was applied, the RF model showed an excellent performance with an AUC of 0.814, F1-score of 0.813, and sensitivity of 0.832. The RF model still showed the best performance, with 0.838 for both the AUC and F1-score, when 12 features were used. When all 20 features were used, the XGBoost model showed the best performance, with 0.851 for both AUC and F1-score. The overall performance of the MetS prediction models improved after applying the SMOTE, with the full XGBoost model showing the best performance (Table 2).

### Key factors in predicting metabolic syndrome

**Fig. 1:**
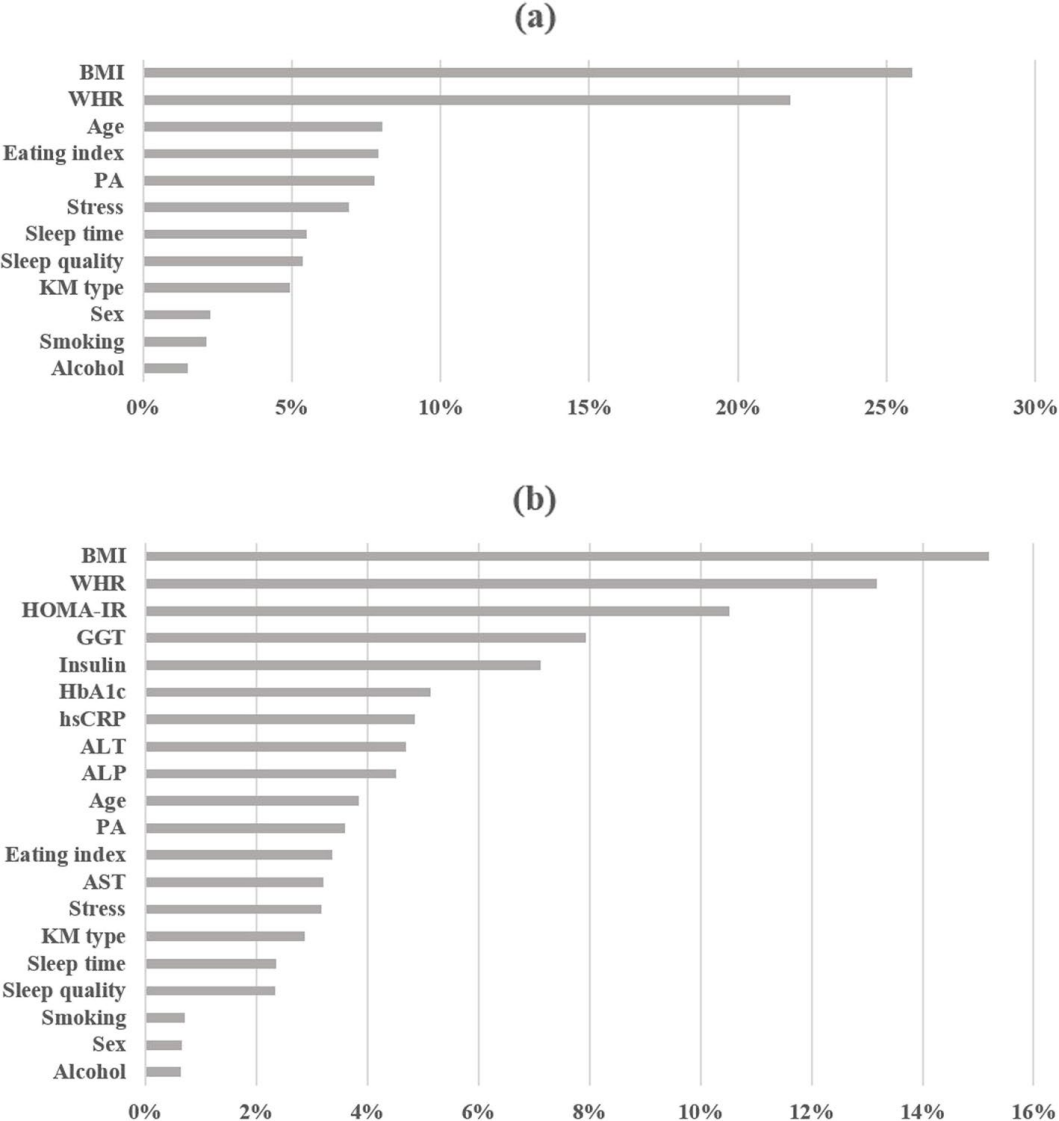
Feature importance in the MetS prediction model. **a** Feature importance when using 12 features; (**b**) Feature importance when using 20 features. Variable importance results when building the model are presented. BMI, body mass index; WHR, waist-to-hip ratio; PA, physical activity; KM type, Korean medicine type; HOMA-IR, homeostatic model assessment for insulin resistance; GGT, gamma-glutamyl transferase; HbAlc, hemoglobin A1c; hsCRP, high sensitivity C-reactive protein; ALT, alanine transaminase; ALP, alkaline phosphatase; AST, aspartate transaminase

Figure 1 shows how each feature influences the prediction of MetS. The key features in models using 12 features were BMI and WHR, with importance of 26 and 22%, respectively (Fig. 1a). Other lifestyle-related features showed relatively weak influence. Despite adding the blood test features, BMI and WHR were still the key features in the models using 20 features, with importance of 15 and 13%, respectively (Fig. 1b). Moreover, the influence of the blood test features seemed higher than the lifestyle-related features. The numerical decreased in the influence of BMI and WHR was due to the increase in the number of features, but these two features were identified as key features in model construction.

## Discussion

The present study applied various machine learning and deep learning techniques to construct MetS prediction models for middle-aged Koreans and verified the performance of the models by changing the number of features (4, 12, and 20) used to construct them. In this process, sex and age were used as the basic features, and the number of features was increased by first including easily measurable anthropometric data, followed by lifestyle-related features obtained through a questionnaire, and lastly, blood test results measured in an invasive method. The results confirmed that the models’ performance improved with the increase in the number of features used and showed the highest scores with 20 features. Among the various models, the RF and XGBoost models showed excellent performances, confirming the importance of BMI and WHR as key features. Moreover, the study demonstrated that data imbalance could be corrected by performing data augmentation with the SMOTE.

The Gaussian NB model showed excellent performance in predicting MetS using sex and anthropometric data (BMI and WHR), while the RF model showed excellent performance in the AUC and F1-score when the lifestyle-related features were included. MetS prediction based on all 20 features without the SMOTE showed moderate. Similar performance results were reported in previous studies [35, 41]. Moreover, in a study that measured lifestyle factors such as smoking status, physical activity, sleep time, shift work, and work-related stress in an Iranian working population to predict MetS using an artificial neural network, the results showed high predictive power with 89% accuracy, 82.5% sensitivity, and 92.2% specificity, significantly better than the traditional logistic regression analysis prediction model [16]. Furthermore, the study stressed the importance of lifestyle factors, such as work-related stress and sleep apnea, in addition to clinical blood markers, for achieving accurate prediction of the MetS status. Moreover, a study on MetS prediction among Koreans with normal weight showed a sensitivity of 0.38–0.42 and an accuracy of 0.71–0.82. The AUC value improved from 0.65 to 0.69 when a Gaussian NB model was applied and genetic data were added to demographic, lifestyle, and clinical data [15]. Such differences in performance between studies are believed to be attributable to differences in the study population, whether only basic clinical data were used when constructing the machine learning models, and differences in the use of genetic or blood test data.

The BMI and WHR proved the most important key features contributing to the MetS model predictive accuracy. In a study on MetS prediction using the Isfahan Cohort Study data of 2107 participants [41], SVM and decision tree-based prediction models were constructed based on various health features, showing sensitivities of 0.774 and 0.758, respectively. The study mentioned that the BMI acted as a key feature. Another study using data of 468 participants from the same cohort found that features such as BMI and WHR were useful indicators of MetS among females [42]. Anthropometric indicators such as BMI and WHR could be easily measured in daily life. Previous studies reported that anthropometric changes in the middle-aged population were a major contributor to MetS prediction, consistent with the findings in the present study. Besides, the present study also examined the influence of lifestyle factors such as eating index, physical activity, sleep time, smoking status, and drinking status [43] as modifiable factors for chronic CVD and KM type, known independent risk factors of MetS [44]. Although, as symptom-based KM type, their contribution to MetS prediction was weak in this study, MetS prediction after adding the lifestyle features showed an adequate level of accuracy. This approach could help identify the risk of MetS through self-diagnosis, so daily life interventional management of MetS could be customized.

Our study also confirmed performance improvement by applying the SMOTE to resolve the data imbalance problem that often occurs when using medical data. A study that used medical data to predict diabetes overcame data imbalance through oversampling with the SMOTE, increasing the sensitivity of probabilistic neural network (from 0.027 to 0.667), decision tree (from 0.215 to 0.726), and Gaussian NB (from 0.721 to 0.776) [38]. In a study that applied the SMOTE to predict heart disease, the extra tree classifier algorithm accuracy improved from 0.833 to 0.926 after the SMOTE was applied [13]. XGBoost performance in our study improved after applying the SMOTE, with sensitivity increasing from 0.662 to 0.859 and accuracy from 0.804 to 0.851 in a model constructed using all 20 features. Moreover, the sensitivity was low, and specificity was high before applying SMOTE. This change was because there were fewer participants in the MetS group than the normal group. However, the specificity decreased slightly and the sensitivity markedly improved when the SMOTE was applied to resolve the data imbalance between the two groups. Considering the characteristics of medical data that frequently show imbalance between groups, the SMOTE for data oversampling could be effective for developing diagnostic approaches, such as MetS prediction.

The present study verified the performance of various machine learning techniques for predicting MetS in middle-aged Koreans, demonstrating that the prediction performance could be improved by data augmentation and increasing the number of features. However, there is potential for further model development due to several limitations. A previous study showed that performance of models constructed to include genetic data was better than those based on clinical data alone [15]. Therefore, improved performance of our models could be expected by adding genetic data. Moreover, while tree-based machine learning models, such as RF and XGBoost, showed excellent performance, the simple 1D-CNN-based deep learning model also performed better than basic statistical analysis methods such as logistic regression or some other machine learning models. Since the present study used a simple deep learning structure, better performance may be expected by applying more advanced deep learning network techniques to the clinical data. The risk factors of MetS analyzed in this study used cross-sectional data; however, cross-sectional data are limited as they do not allow accurate analysis of causal relationship between the disease onset and its risk factors. Because the sample size in this study is small, this model may be less accurate with a larger sample. In order to avoid optimally biased performance estimates in machine learning analysis, it is important to separate training data and test data or to have a sufficient number of samples [45]. Considering this, further studies using large-scale data are needed. Finally, the MetS group in present study included pre-MetS status, satisfying at least two criteria, taking into account the low prevalence of MetS in the study participants. However, the present study was the first to investigate the effect of increasing the number of used features by machine learning techniques to predict MetS. The machine learning-based models showed good performance in predicting MetS, particularly the tree-based RF and XGBoost models.

Despite these limitations, the present study could help the middle-aged population lower the risk of aging-related chronic diseases such as MetS through routine healthcare and assessment of easily modifiable lifestyle factors. Moreover, a strength of the study was its multi-faceted MetS management models that compared the performance through stepwise inclusion of daily life prediction factors such as weight, lifestyle, and data from medical institutions such as blood test results.

## Conclusions

The present study used anthropometric, lifestyle, and blood test features to compare the performance of MetS prediction models in middle-aged Koreans. Among these MetS prediction models, the tree-based machine learning ones showed high accuracy in identifying participants with MetS. The models’ performance improved when the number of features was increased, and the SMOTE was applied. The anthropometric features BMI and WHR were identified as more important features for MetS prediction in this middle-aged population than lifestyle or blood test features. Early diagnosis of MetS is important, requiring a multidimensional approach that includes self-administered questionnaire, anthropometric, and biochemical measurements.

## Data Availability

The data supporting the conclusions of this article are available with approval from the Korea Medicine Data Center (KDC) of the Korea Institute of Oriental Medicine but restrictions apply to the availability of these data, which were used under license for the current study, and so are not publicly available. Data are however available from the authors upon reasonable request and with permission of the KDC (https://kdc.kiom.re.kr).

## Abbreviations

1D-CNN: 1D convolutional neural network
ALP: Alkaline phosphatase
ALT: Alanine transaminase
AST: Aspartate transaminase
AUC: Area under the receiver operating characteristic curve
BMI: Body mass index
CVD: Cardiovascular disease
Gaussian NB: Gaussian naïve bayes classifier
HbA1c: Hemoglobin A1c
HOMA-IR: Homeostatic model assessment for insulin resistance
hsCRP: High-sensitivity C-reactive protein
KDCC: Korean Medicine Daejeon Citizen Cohort
KM: Korean medicine
KNN: K-nearest neighbor
MetS: Metabolic syndrome
MLP: Multi-layer perceptron
NCEP-ATP III: National Cholesterol Education Program-Adult Treatment Panel III
RF: Random forest
SMOTE: Synthetic minority oversampling technique
SVM: Support vector machine
WHR: Waist-to-hip ratio
XGBoost: eXtreme gradient boosting
GGT: Gamma-glutamyl transferase
